# Bilateral Spontaneous Supraspinatus Tendon Rupture Under Prolonged BRAF/MEK Targeting Treatment in a Melanoma Patient

**DOI:** 10.7759/cureus.50567

**Published:** 2023-12-15

**Authors:** Dimitrios Bafaloukos, Ioanna Gazouli, Christos Koutserimpas, Pantelis D Skarlos, George Samonis

**Affiliations:** 1 Department of Medical Oncology, Metropolitan Hospital, Athens, GRC; 2 Department of Orthopedics and Traumatology, 251 Air Force General Hospital, Athens, GRC; 3 Department of Anatomy, National and Kapodistrian University of Athens, Athens, GRC; 4 Department of Radiation Oncology, Metropolitan Hospital, Athens, GRC; 5 Department of Medicine, University of Crete, Heraklion, GRC

**Keywords:** rotator cuff tear, side effects, tendon rupture, trametinib, braf/mek targeting, braf mutation, melanoma

## Abstract

The B-Raf proto-oncogene, serine/threonine kinase (BRAF)/ mitogen-activated protein kinase kinase (MEK) targeting agents have become the treatment of choice for BRAF-mutated melanoma during the last decade. However, it is possible that some long-term adverse events of these drugs have not yet been reported. A case of bilateral spontaneous, non-traumatic, supraspinatus tendon rupture in a 65-year-old Caucasian male suffering metastatic melanoma under prolonged and successful combination treatment with dabrafenib plus trametinib is presented. These damages could not be attributed to some other probable cause. The ruptured tendons were promptly restored arthroscopically. Oncologists should remain vigilant for the early detection of potential side effects of BRAF/MEK targeting agents that have not been systematically recorded yet but may appear and affect patients in the long run.

## Introduction

Medication-associated tendinopathies and tendon ruptures have been described under treatment with several pharmaceutical agents, such as corticosteroids, statins, quinolones, and aromatase inhibitors [1, 2]. Most frequently, the Achilles tendon is affected [3-5]. Several underlying mechanisms facilitating this clinical entity have been proposed, such as local hypoxia and impaired fibroblast activity, in combination with predisposing patient-related factors, including age and gender, as well as overuse caused by exercise and/or vigorous physical activity [6, 7].

Novel B-Raf proto-oncogene, serine/threonine kinase (BRAF)/ mitogen-activated protein kinase kinase (MEK) targeting agents have been incorporated in the standard of care of BRAF mutated melanoma since 2011 and improved dramatically the therapeutic effects, inducing high objective response rates, and prolonging patient survival [8]. Most side effects of these agents are known and have been meticulously studied. However, despite the fact that these drugs have been in use for more than a decade, it is possible that some adverse events due to their use have not been observed yet. Hence, some long-term side effects are yet to be reported. The most frequent adverse events of BRAF/MEK targeting agents include pyrexia, skin rash, and hepatic enzyme elevation. Musculoskeletal complications, mainly muscle and joint aches, are reported at a low rate, affecting 1-2% of treated patients [8]. BRAF/MEK inhibitors have not yet been associated with tendinopathies.

A case of spontaneous, non-traumatic, bilateral supraspinatus tendon rupture, occurring in a 65-year-old Caucasian male under prolonged treatment with dabrafenib plus trametinib for a stage IV, BRAF mutated melanoma, is presented.

## Case presentation

The 65-year-old patient was first examined 11 years ago due to a melanoma relapse on the right lateral chest wall. The primary lesion was located at his frontal abdominal surface and had been surgically removed eight years earlier. It has been characterized as stage IB, pT2aN0M0 melanoma, with a Breslow depth of 1.45mm and Clark stage IV. The tumor was BRAF V600E mutated. The initial sentinel lymph node biopsy was negative. No adjuvant treatment had been administered.

Due to disease relapse, he underwent thorough clinical, laboratory, and imaging examinations for staging his disease. No other suspicious lesions had been recorded. Hence, systematic targeted treatment with dabrafenib (oral BRAF inhibitor, 150mg twice daily) and trametinib (oral MEK inhibitor, 2mg once daily) was initiated in the context of a clinical trial protocol. The patient enjoyed an impressive complete disease remission. Ever since the trial's termination, the dabrafenib/trametinib regimen was consistently administered for more than 10 years under close medical surveillance. To date, there are no clinical or imaging findings suggesting disease recurrence.

After completing 130 months under treatment uneventfully, he started complaining of pain and limited movement ability of his right shoulder and lesser similar symptoms of his left shoulder. Upon clinical examination, the patient experienced pain while lifting and lowering his arm, as well as at rest during the night. The Jobe test was positive on both sides (weakness and pain at requested shoulder abduction and internal rotation). Patient's remaining medical history was unremarkable, and he was not receiving any other medications except dabrafenib and trametinib at that moment. He was active with excellent performance status, but he did not report any heavy physical activity or overhead activities.

**Figure 1:**
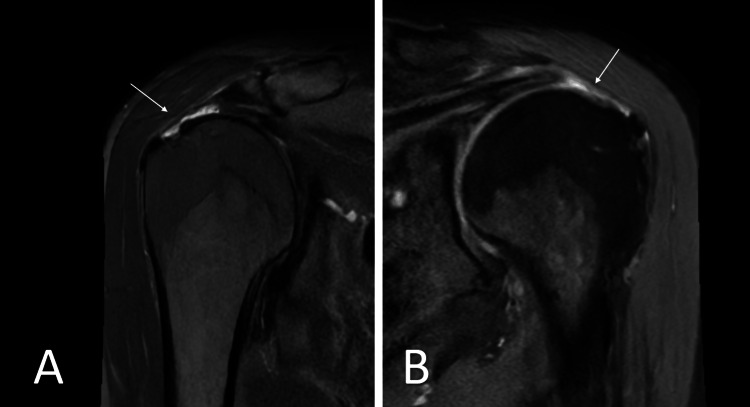
Magnetic resonance imaging (MRI) of both shoulders A) MRI T2 weighted images with fat suppression of the right shoulder in the coronal plane revealing the rupture of the supraspinatus tendon (arrow) with a retraction of approximately 2cm. B) MRI protein density weighted images in the coronal plane of the left shoulder showing the supraspinatus tendon tear with a retraction of approximately 1.8 cm.

Magnetic resonance imaging (MRI) revealed rupture of the supraspinatus tendon with approximately 2cm retraction on both sides. Both tendons had degenerative signs, such as calcific tendinopathy, as well as signs of subacromial impingement (Figure 1)

Biopsy of the affected tendons' areas revealed local trichoid vessel congestion and mild reactive lesions on the synovial membrane but was negative for inflammation immunohistochemical markers.

Both tendons were arthroscopically repaired with the use of two suture anchors. The patient had an uneventful recovery, while the dabrafenib/trametinib treatment was continued after the mandatory one-month interval during the surgical intervention. Twelve months postoperatively, the patient has active shoulder abduction up to 168 degrees on the right and 160 on the left side. He does not complain of any shoulder pain, and the Jobe test is negative.

Since he had not been receiving any other drugs that could have caused the tendon ruptures for a long time before the incident, it is highly probable that the tendons' ruptures may be an adverse event of perpetuated dabrafenib and trametinib treatment.

## Discussion

A meticulous search of the literature indicates that this is the first case of tendon rupture associated with dabrafenib/trametinib combination or any BRAF/MEK inhibitor combination.

A case of multifocal tendon rupture in a 58-year-old male, under treatment with nivolumab plus ipilimumab for metastatic melanoma, has been recently described [9], but BRAF-directed treatment has not been suspected of inducing tendinopathies to date.

Both nivolumab and ipilimumab are potent immune checkpoint inhibitors, successfully applied in metastatic melanoma treatment, acting in a totally different manner from targeted treatment with dabrafenib/trametinib. While dabrafenib/trametinib block proteins crucial to cellular proliferation, nivolumab and ipilimumab enhance T-lymphocyte cytotoxic activity by blocking immune suppressive receptors expressed on the T cell surface, known as programmed death receptor 1 (PD-1) and cytotoxic T-lymphocyte associated protein 4 (CTLA-4), respectively [8, 9]. Ipilimumab and nivolumab combination could lead to tendonitis and tendon rupture of autoimmune etiology, whereas there is no known mechanism for dabrafenib/trametinib-associated tendon damage.

Medication-associated tendon rupture has been attributed to local hypoxia, frequently affecting critical tendon areas where blood flow is limited due to relevant anatomy [1, 2]. Impaired metabolism and cell growth of tendon fibroblasts, together with increased matrix proteolytic activity and inhibition of tenocyte translocation to the site of tendon injury, are also among the proposed underlying mechanisms, as indicated by in vitro experiments [10, 11]. Indeed, tendon degeneration has also been described in vivo in mouse models after quinolone treatment [12, 13]. It has to be mentioned, though, that tendon rupture is not induced merely by the associated medications, as predisposing factors, such as female gender, older age, renal insufficiency, and hemodialysis, may be the basis of this damage, often in combination with vigorous physical activity [1, 5]. Hence, although other factors may play an important role in tendon rapture, such as patients' age (the present patient was 65 years old), this report draws clinical attention to patients needing BRAF inhibitor treatment, especially those with coexisting tendon degeneration.

At a microscopic level, collagen fiber disarrangement, hyaline or myxomatous degeneration, and increased metalloproteinase activity, as well as focal necrosis and degenerative vacuoles disrupting healthy tendon structure, have been reported in both humans and mouse models receiving corticosteroids and/or quinolones [14-19].

In the case presented here, trichoid congestion and synovial membrane reaction were described in the affected tendons specimen, with no signs of inflammation, while inflammatory markers were not observed.

## Conclusions

Targeted treatment against BRAF-mutated melanoma has changed the prognosis for thousands of metastatic melanoma patients. In most cases, treatment is continued until disease relapse or progression or unacceptable toxicity, as there is no way to guarantee safe withdrawal without exposing the patient to increased relapse risk. Nevertheless, long-term adverse events associated with novel melanoma treatments may only now start to appear and be reported. Physicians should remain vigilant for early detection and offer treatment against adverse reactions of BRAF targeting agents that have not been systematically recorded yet but may affect patients in the long run. Additionally, a thorough investigation has to be conducted to understand further the pathophysiology and the prevention of this rare but significant side effect.
